# [EMmim][NTf_2_]—a Novel Ionic Liquid (IL) in Catalytic CO_2_ Capture and ILs’ Applications

**DOI:** 10.1002/advs.202205352

**Published:** 2022-11-23

**Authors:** Xin He, Yangyan Gao, Yunlei Shi, Xiaowen Zhang, Zhiwu Liang, Riguang Zhang, Xingfei Song, Qinghua Lai, Hertanto Adidharma, Armistead G. Russell, Eric G. Eddings, Weiyang Fei, Fangqin Cheng, Shik Chi Edman Tsang, Jianji Wang, Maohong Fan

**Affiliations:** ^1^ Departments of Petroleum and Chemical Engineering University of Wyoming Laramie WY 82071 USA; ^2^ College of Materials and Chemistry & Chemical Engineering Chengdu University of Technology Chengdu 610059 P. R. China; ^3^ College of Environmental & Resource Science of Shanxi University Taiyuan 030001 P. R. China; ^4^ School of Chemistry and Chemical Engineering Henan Normal University Xinxiang Henan 453007 P. R. China; ^5^ College of Chemistry and Chemical Engineering Hunan University Changsha 410082 P. R. China; ^6^ Key Laboratory of Coal Science and Technology of Ministry of Education and Shanxi Province Taiyuan University of Technology Taiyuan Shanxi 030024 P. R. China; ^7^ Key Laboratory on Resources Chemicals and Materials of Ministry of Education Shenyang University of Chemical Technology Shenyang 110142 P. R. China; ^8^ School of Civil and Environmental Engineering Georgia Institute of Technology Atlanta GA 30332 USA; ^9^ Department of Chemical Engineering University of Utah Salt Lake City UT 84112 USA; ^10^ State Key Laboratory of Chemical Engineering Department of Chemical Engineering Tsinghua University Beijing 100084 P. R. China; ^11^ Department of Chemistry University of Oxford Oxford OX1 3QR UK

**Keywords:** absorption and desorption, catalyst, CO_2_ capture, ionic liquid, monoethanolamine

## Abstract

Ionic liquids (ILs) have been used for carbon dioxide (CO_2_) capture, however, which have never been used as catalysts to accelerate CO_2_ capture. The record is broken by a uniquely designed IL, [EMmim][NTf_2_]. The IL can universally catalyze both CO_2_ sorption and desorption of all the chemisorption‐based technologies. As demonstrated in monoethanolamine (MEA) based CO_2_ capture, even with the addition of only 2000 ppm IL catalyst, the rate of CO_2_ desorption—the key to reducing the overall CO_2_ capture energy consumption or breaking the bottleneck of the state‐of‐the‐art technologies and Paris Agreement implementation—can be increased by 791% at 85 °C, which makes use of low‐temperature waste heat and avoids secondary pollution during CO_2_ capture feasible. Furthermore, the catalytic CO_2_ capture mechanism is experimentally and theoretically revealed.

## Graphical abstract

A uniquely designed and synthesized ionic liquid (IL) is first used as a homogeneous catalyst for monoethanolamine (MEA) based CO_2_ capture. Only 2000 ppm IL catalyst can universally catalyze both CO_2_ sorption and desorption of all the chemisorption‐based technologies. Furthermore, the catalytic CO_2_ capture mechanism is experimentally and theoretically revealed.

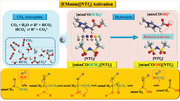

## 1 Introduction

Carbon dioxide (CO_2_) capture is critical not only because of its close connection with climate change according to Paris Climate Accord ^[^ ^1^, ^2^, ^3^ ^]^ but also because of its increasing importance as a material and fuel synthesis resource.^[^ ^4^, ^5^, ^6^ ^]^ Thus, CO_2_ capture is very important.^[^ ^7^, ^8^ ^]^ Chemisorption is one of the most important methods for CO_2_ capture.^[^ ^9^, ^10^, ^11^ ^]^ The fundamental challenge of chemisorption‐based technologies is the slow absorption and desorption reaction kinetics, especially the latter one, which leads to the need for CO_2_ desorption at >100 °C.^[^ ^12^ ^]^ Consequently, excessive energies are needed to vaporize a large amount of liquid water during CO_2_ desorption operation and condense the same amount of water vapor prior to CO_2_ sorption during cyclic CO_2_ sorption and desorption. Also, severe corrosion and sorbent degradations could result in secondary environmental and health issues, especially when organic amines are used as sorbents.

Thus, is there a way to overcome the fundamental and the associated challenges by desorbing CO_2_ or regenerating spent sorbents at temperatures less than 100 °C? This is the pivotal to making use of abundant low quality or waste heats for CO_2_ desorption feasible, while desired CO_2_ desorption rates are achievable. Fan et al.^[^ ^13^ ^]^ reported that 20 000 ppm nanostructured TiO(OH)_2_ as a heterogeneous catalyst can increase CO_2_ desorption rate of MEA‐based CO_2_ capture over 4000% at 88 °C . However, to date, homogeneous catalysis has not been utilized for CO_2_ capture.

After a long time of study, a homogeneous CO_2_ capture catalyst—a new ionic liquid (IL), [CH_3_COOCH_2_mim][NTf_2_], denoted as [EMmim][NTf_2_]—was discovered. The IL, used as a CO_2_ capture catalyst instead of a sorbent, is not only very effective for catalyzing CO_2_ desorption but also CO_2_ sorption. It should be emphasized that [EMmim][NTf_2_] is used as a transformative catalyst instead of a sorbent for CO_2_ capture ^[^ ^14^ ^]^ in this work.

## 2 Results and Discussion

### 2.1 Effect of the Synthesized IL Catalyst

Detailed procedure for preparing [EMmim][NTf_2_], the first IL and homogeneous catalyst for CO_2_ capture, is provided in the Materials and Methods part. A schematic drawing of this CO_2_ absorption and desorption experimental setup is shown in Figure S1 (Supporting Information). Schematic diagram for synthesizing the IL and its characteristic results are provided in Figure S2 (Supporting Information). The optimal dosage of this catalyst for accelerating CO_2_ desorption, the key step for reducing overall CO_2_ capture energy consumption, is 2000 ppm according to Figure S3 (Supporting Information).

**Figure 1:**
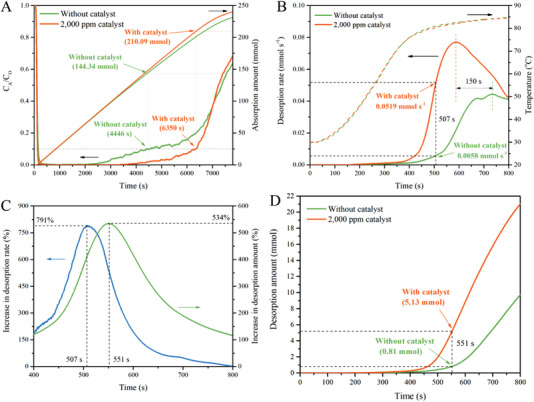
Catalytic effects of [EMmim][NTf_2_] on CO_2_ absorption and desorption. A) Uncatalyzed and catalyzed CO_2_ absorption profiles of 20% MEA sorbent; B) effects of CO_2_ desorption rates with and without uses of the catalyst; C) the percentage increases in CO_2_ desorption rate and desorption amount with the use of catalyst; D) effect of catalyst on the quantities of desorbed CO_2_.

**Figure** **1** exhibits the CO_2_ absorption and desorption performances of 20 wt% monoethanolamine (MEA) solution catalyzed by 2000 ppm [EMmim][NTf_2_]. [EMmin][NTf_2_] can promote both CO_2_ absorption and desorption. Capturing 90% CO_2_ is targeted by the U.S. Department of Energy, and thus the period with 90% CO_2_ capture efficiency is defined as effective absorption time. According to Figure 1A, 20 wt% MEA sorbent without catalyst can absorb only 144.34 mmol CO_2_ within the shorter effective absorption time of 4446 s, while the sorbent with 2000 ppm IL catalyst can absorb 210.09 mmol CO_2_, and extend effective absorption time to 6350 s, which are 45.55% and 42.83% absorption improvements, respectively, compared to the sorbent without catalyst.

Variations of desorption with time with and without uses of catalyst are illustrated in Figure 1B. The time needed for reaching the maximum desorption rate with the use of [EMmim][NTf_2_] is shortened by 150 s in comparison to the time required for CO_2_ desorption without the use of the IL catalyst. Meanwhile, the peak desorption rate improvement is 175%. The increases in desorption rate and the desorbed CO_2_ amount obtained with [EMmim][NTf_2_] can reach as high as 791% at 507 s and 534% at 551 s (Figure 1B–D), respectively. The quantities of CO_2_ desorbed from uncatalytic and catalytic solutions are 18.7 and 28.2 mmol, respectively. When the absorption time is 7000 s or the outlet CO_2_ concentrations of both uncatalyzed and catalyzed CO_2_ absorption are 3.2 vol% (Figure S4, Supporting Information), use of [EMmim][NTf_2_] can increase the total desorbed CO_2_ amount by 88.90%. It should be noted that dosage of [EMmim][NTf_2_] is only 2000 ppm—the lowest dosage for achieving such significant CO_2_ capture catalysis effect. To the best knowledge, such a low catalyst dosage has not been reported for achieving such a significant CO_2_ capture. Also, as a homogeneous catalyst, [EMmim][NTf_2_] is easy to use. Therefore, [EMmim][NTf_2_] is quite effective for catalyzing both the CO_2_ absorption and desorption processes.

### 2.2 Stabilities of the Catalytic CO_2_ Capture System

**Figure 2:**
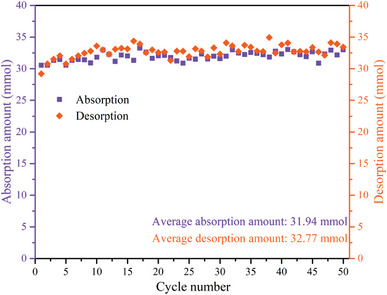
Evaluation of stability of [EMmim][NTf_2_] as a catalyst for MEA based CO_2_ capture.

Stability characteristic of [EMmim][NTf_2_] for CO_2_ capture was studied with 50 cyclic tests, and the results are shown in **Figure** **2** and Figure S5 (Supporting Information). There is no apparent change in both quantities of absorbed and desorbed CO_2_. The average CO_2_ capture working capacities within the 50 cyclic tests are 31.94 and 32.77 mmol for absorption and desorption amounts, respectively. As shown in Figure S5C (Supporting Information), FT‐IR spectra of the regenerated MEA solution confirm that no change in the structure of MEA molecules was observed after 50 cycles of the tests. Thus, both MEA and the [EMmim][NTf_2_] are stable. Moreover, [EMmim][NTf_2_] can thermally be stable at as high as 300 °C as shown in Figure S2F (Supporting Information).

### 2.3 Mechanism

#### 2.3.1 Experimental Study of the Reaction Mechanisms

**Figure 3:**
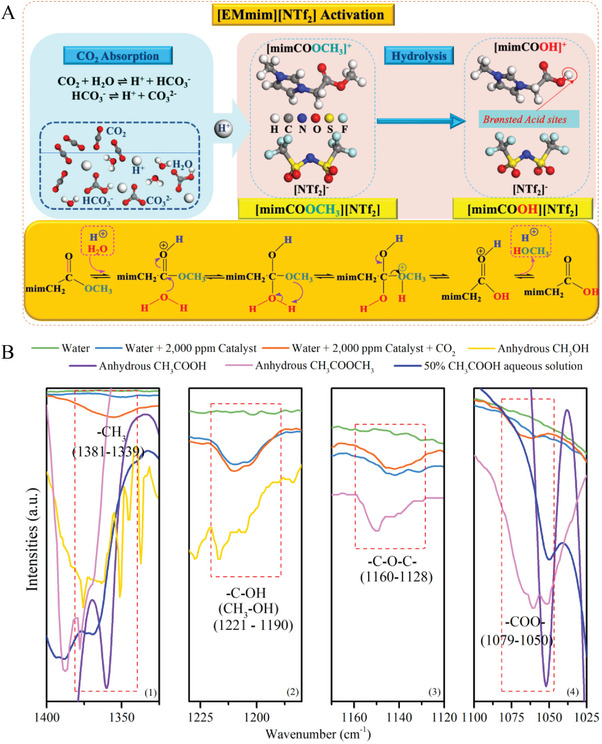
The [EMmim][NTf_2_] catalyst activation process via hydrolysis in CO_2_ absorbing system. A) The schematic representation of the [EMmim][NTf_2_] catalyst and the hydrolysis activation process. B) FT‐IR spectra of water, water +2000 ppm catalyst with or without additions of CO_2_, anhydrous CH_3_OH and CH_3_COOH as well as CH_3_COOCH_3_, and 50% CH_3_COOH aqueous solution. 5.18 mmol CO_2_ was bubbled into the water + 2000 ppm Catalyst + CO_2_ system with a simulated flue gas containing 10 vol% CO_2_, 10 vol% O_2_, and 80 vol% N_2_ with a total flow rate of 500 mL min^−1^ in 20 min.

**Figure 4:**
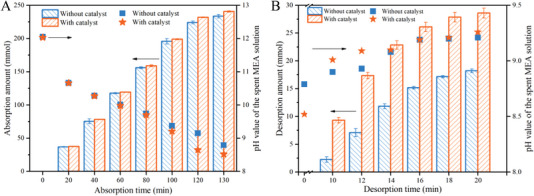
Changes of the quantities of CO_2_ absorbed and desorbed, and pH values of the uncatalytic and catalytic solutions with A) absorption times and B) desorption times.

The molecular structure of the IL, [EMmim][NTf_2_] is shown in Figure S2A (Supporting Information). After CO_2_ absorption, the pH of the capture system decreased from 12.01 to 8.52 (**Table** **1**), indicating the increase of H^+^ concentration and promotion of the hydrolysis of the ‐COOCH_3_ in [EMmim][NTf_2_] into —COOH, which is need for catalyzing CO_2_ desorption. Thus, the activation of [EMmim][NTf_2_] is accompanied with the enhancement of the acidity of [EMmim][NTf_2_] after CO_2_ absorption, as illustrated in **Figure** **3**A. The FT‐IR spectra of water, and the 2000 ppm [EMmim][NTf_2_] aqueous solution with or without CO_2_ shown in Figure 3B further confirm this mechanism. Anhydrous CH_3_OH and CH_3_COOCH_3_ as well as CH_3_COOH and 50% CH_3_COOH solutions were used to identify the functional groups in the catalytic solutions with or without additions of CO_2_. The bands around 1381–1339 cm^−1^ in the catalytic solutions [Figure 3B (1)] are assigned to the —CH_3_ group in the IL catalyst (—COOCH_3_), observed in anhydrous CH_3_OH, CH_3_COOCH_3_ and CH_3_COOH and 50% CH_3_COOH solutions. With the increase in the introduced CO_2_ in the solution, hydrolysis of ‐COOCH_3_ is enhanced, leading to increasing the concentration of —CH_3_ or CH_3_OH in the catalytic solution as illustrated in Figure 3A. Bands with wavenumbers between 1221–1190 cm^−1^ [Figure 3B (2)], detected in anhydrous CH_3_OH as well, confirm the formation of CH_3_OH with an enhanced hydrolysis process with CO_2_. As concentration of —COOCH_3_ decreases, the —C—O—C peak [1160–1128 cm^−1^, Figure 3B (3)] in the catalytic solution with the addition of CO_2_ was weaker than that of the solution without addition of CO_2_ according to the reference peaks of anhydrous CH_3_COOCH_3_. Consequently, more —COO— or —COOH formation or more —COOCH_3_ hydrolysis, was detected within 1079–1050 cm^−1^ [Figure 3B (4)], which are concluded from the observations of peaks of anhydrous CH_3_COOCH_3_ and CH_3_COOH, and 50% CH_3_COOH solution. Also, the acidity of the [EMmim][NTf_2_] was verified via the measurement of the pH (6.19) of the solution resulting from the addition of 0.2 g [EMmim][NTf_2_] to 80 g H_2_O (Table 1). The FT‐IR spectra of fresh catalytic solution, catalytic solutions after the 1^st^ absorption and the 1^st^ absorption–desorption runs are displayed in Figure S6 (Supporting Information). The differences in the changes of the quantities of CO_2_ absorbed and desorbed, and pH values of the uncatalytic and catalytic solutions with absorption and desorption times as shown in **Figure** **4** result from the use of the catalyst. The uncatalytic solution and catalytic solution both absorbed 232 mmol CO_2_ at 130 and 120 min, respectively. However, the pH value (8.65) of the catalytic solution is lower than that (8.79) of the uncatalytic solution, which is another direct evidence of hydrolysis of ‐COOCH_3_ in the IL into —COOH, a key functional group in catalyzing the subsequent CO_2_ desorption.

**Table 1:** pH values of aqueous IL solutions and mMEA solutions

Sample	pH values
80 g H_2_O	6.37
80 g H_2_O + 0.2 g [EMmim][NTf_2_]	6.19
20 g MEA + (80 g H_2_O + 0.2 g [EMmim][NTf_2_])	12.01
20 g MEA + 80 g H_2_O	12.04
MEA without catalyst after the 1^st^ abs^1^.	8.79
MEA with [EMmim][NTf_2_] catalyst after the 1^st^ abs^2^.	8.52
MEA without catalyst after the 1^st^ cyclic abs.‐des^3^.	9.47
MEA with [EMmim][NTf_2_] catalyst after the 1^st^ cyclic abs.‐des^4^.	9.46
MEA with [EMmim][NTf_2_] catalyst after 50 cycles of abs.‐des.	9.34

Note:^1, 2^ 1^st^ abs.: solution received after the 2 h 10 min sorption. ^3,4^ 1^st^ cyclic abs.‐des.: solution obtained after the first cyclic test.

**Figure 5:**
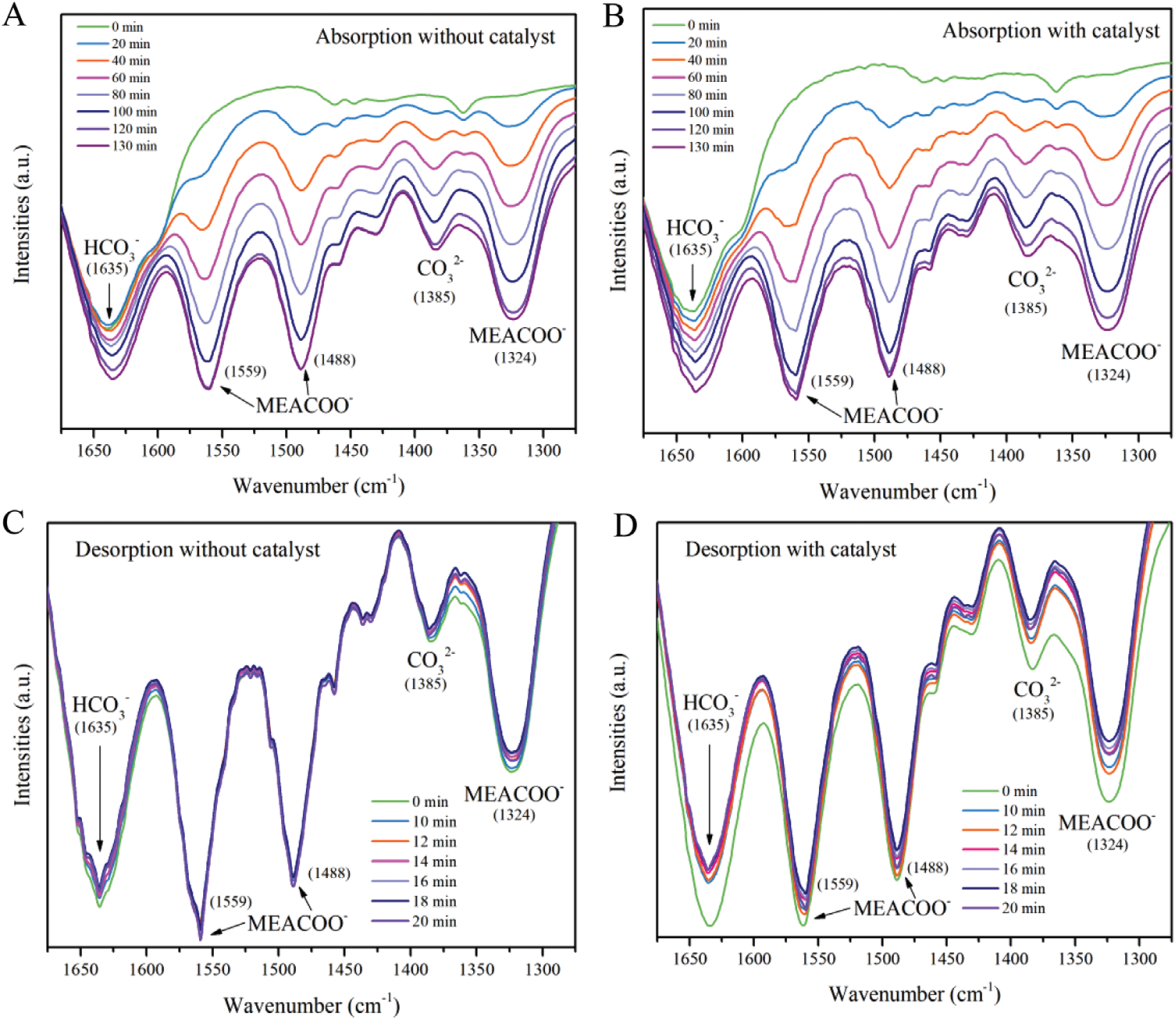
FT‐IR spectra of the catalytic and uncatalytic solutions during CO_2_ absorption and desorption at different times. A) Uncatalyzed CO_2_ absorption; B) catalyzed CO_2_ absorption; C) uncatalyzed CO_2_ desorption; D) catalyzed CO_2_ desorption.

The catalytic effect of [EMmim][NTf_2_] at different absorption and desorption times is observed through FT‐IR spectra, as shown in **Figure** **5**. The bands at 1324 cm^−1^ (stretching of N—COO^−^), 1488 cm^−1^ (symmetric stretching of COO^−^) and 1559 cm^−1^ (asymmetric stretching of COO^−^) are ascribed to the MEACOO^−^,^[^ ^15^ ^]^ while peaks at 1385 and 1635 cm^−1^ are assigned to CO_3_^2−^ and HCO_3_^−^, respectively.^[^ ^15^, ^16^ ^]^ Intensities of peaks of HCO_3_^−^ in the catalytic MEA solution during CO_2_ absorption (Figure 5B) increase faster than those of the uncatalytic ones (Figure 5A). Moreover, peak intensities of MEACOO^−^ and CO_3_^2−^ for the catalytic MEA solutions at the end of CO_2_ absorption (7800 s or 130 min) are stronger than those of the uncatalytic one. Differences are more obvious for the desorption tests, as shown in Figure 5C,D. Peak intensities of HCO_3_^−^ in the uncatalytic MEA solutions decrease slowly with the continuous CO_2_ desorption, and the intensities of CO_3_^2−^ and MEACOO^−^ peaks and thus the concentrations of CO_3_^2−^ and MEACOO^−^ barely change, in spite of the subsequent slightly noticeable variations. However, peak intensities of HCO_3_^−^, CO_3_^2−^and MEACOO^−^ of the catalytic MEA solutions decrease considerably fast with time during CO_2_ desorption, especially in the initial 10 min. The experimental observations of the changes in concentrations of intermediates with FT‐IR spectra confirm the significant catalytic effect of the [EMmim][NTf_2_].

#### 2.3.2 Uncatalyzed and Catalyzed CO_2_ Sorption and Desorption Pathways as well as Their Essential Differences

**Figure 6:**
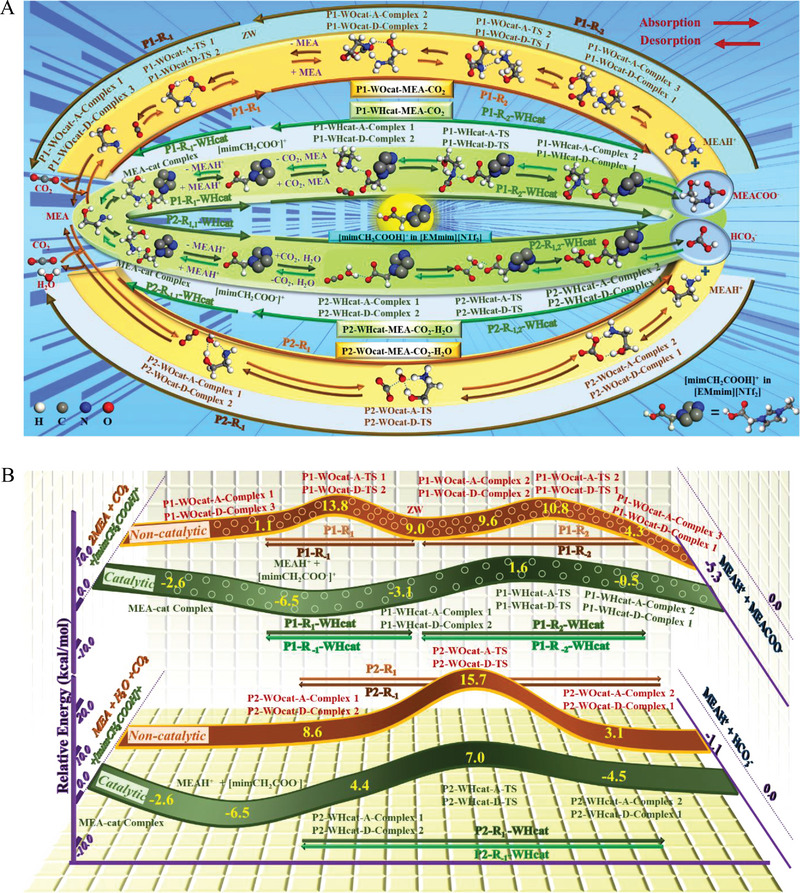
DFT based noncatalytic and catalytic CO_2_ capture reaction pathways and energy profiles. A) Pathways. B) Energy profiles.

The density functional theory (DFT) calculations in this research have been performed by using the Gaussian09 package.^[^ ^17^ ^]^ The reaction processes with and without uses of the catalyst are shown vertically and horizontally in **Figure** **6**A, respectively, while the corresponding energy changes for each step are provided in Figure 6B. Also, the calculation results reveal the highest energy barriers in CO_2_ absorption and desorption processes (especially CO_2_ sorption) and how they can be lowered with the catalyst [EMmim][NTf_2_].

When catalyst is not used, there are two possible CO_2_ capture pathways, P1‐WO_cat_‐MEA‐CO_2_ (pathway without presences of the catalyst and H_2_O) and P2‐WO_cat_‐MEA‐CO_2_‐H_2_O (pathway without presence of the catalyst but with the presence of H_2_O) according to zwitterion (ZW) and bicarbonate (HCO_3_^−^) formation mechanisms.^[^ ^18^, ^19^ ^]^ The step reactions in P1‐WO_cat_‐MEA‐CO_2_ shown in Figure 6A include

(P1‐R_1_) $${\mathrm{CO}}_{2}+\mathrm{MEA}↔{\mathrm{MEA}}^{+}{\mathrm{COO}}^{-}\left(\mathrm{ZW}\right)$$

(P1‐R_2_) $${\mathrm{MEA}}^{+}{\mathrm{COO}}^{-}+\mathrm{MEA}↔{\mathrm{MEACOO}}^{-}+\mathrm{MEA}H^{+}$$

The ring configuration of MEA shown in Figure S7 (Supporting Information) is the most stable structure. As shown in Figure 6B, the Gibbs free energy of reaction for the CO_2_ absorption process consisting of P1‐R_1_ and P1‐R_2_ in P1‐WO_cat_‐MEA‐CO_2_ is exothermic by 5.3 kcal mol^−1^. Furthermore, as shown in Figure S8 (Supporting Information), the calculated Gibbs free energy of reaction and activation energies in the forward reaction or CO_2_ absorption and reverse reaction or CO_2_ desorption of P1‐WO_cat_‐MEA‐CO_2_, P1‐R_1_ and P1‐R_2_, as well as P1‐R_‐1_ and P1‐R_‐2_, respectively, are very close to those in the literatures.^[^ ^20^, ^21^, ^22^, ^23^, ^24^, ^25^, ^26^ ^]^ Thus, the computational methods are effective. Special attention should be paid to P1‐R_‐1_ because it is identified as the rate‐determining step of CO_2_ desorption for P1‐WO_cat_‐MEA‐CO_2_ due to its high Gibbs free energy of reaction (6.5 kcal mol^−1^). According to the energetic span model of Kozuch and Shaik,^[^ ^27^ ^]^ the apparent activation energy for CO_2_ absorption and desorption are 13.8 and 19.6 kcal mol^−1^, respectively.

For the HCO_3_^−^ formation mechanisms,^[^ ^18^, ^19^ ^]^ P2‐WO_cat_‐MEA‐CO_2_‐H_2_O, the HCO_3_^−^ generation reactions probably are ^[^ ^28^, ^29^, ^30^ ^]^

(P2‐R_1_) $${\mathrm{CO}}_{2}+\mathrm{MEA}+H_{2}O↔{\mathrm{HCO}}_{3}^{-}+\mathrm{MEA}H^{+}$$

(P2‐R_2‐1_) $${\mathrm{CO}}_{2}+\mathrm{MEA}↔{\mathrm{MEA}}^{+}{\mathrm{COO}}^{-}$$

(P2‐R_2‐2_) $${\mathrm{MEA}}^{+}{\mathrm{COO}}^{-}+H_{2}O\to {\mathrm{HCO}}_{3}^{-}+\mathrm{MEA}H^{+}$$

The reaction energy values of P2‐R_1_ and P2‐R_2_ were calculated and compared in Figure S9 (Supporting Information). P2‐R_1_ is the preferred pathway because of its lower Gibbs free energy (7.1 kcal mol^−1^) for activation among all the steps of P2‐R_1_ and P2‐R_2_. Thus, Figure 6 only shows P2‐R_1_, denoted as P2‐R_1_‐WO_cat_‐MEA‐CO_2_‐H_2_O, and its step reactions as well as the associated energy data.

Then, the assured computational methods were applied to explain how the catalyst [EMmim][NTf_2_] can overcome the challenge of chemisorption‐based CO_2_ capture via significant acceleration of both CO_2_ absorption and CO_2_ desorption with catalyst in P1‐WH_cat_‐MEA‐CO_2_ (pathway with the catalyst but without H_2_O) and P2‐WH_cat_‐MEA‐CO_2_‐H_2_O (pathway with both catalyst and H_2_O). [mimCH_2_COOH]^+^ as a Brønsted acid is the core of the catalytic CO_2_ capture technology, thus, [mimCH_2_COOH]^+^ instead of [EMmim][NTf_2_] is used for modeling the catalyst. In Figure 6, it clearly shows that [mimCH_2_COOH]^+^ in [EMmim][NTf_2_] readily transfers the carboxyl group proton to MEA to form the complex of [mimCH_2_COO^−^]^+^ and MEAH^+^, denoted as MEA‐cat Complex, which is barrierless and exothermic by 2.6 kcal mol^−1^. And it is found that the individual [mimCH_2_COOH]^+^ is effective than the whole MEA‐cat Complex in P1‐WH_cat_‐MEA‐CO_2_, as shown in Figure S10 (Supporting Information). Reactions in the catalyzed desorption pathway are in way of:

(P1‐R_‐2_‐WH_cat_) $$\begin{array}{ccc} & & \mathrm{MEACO}O^{-}+{\left[\mathrm{mimC}H_{2}\mathrm{COOH}\right]}^{+}\to \mathrm{MEA}\\ & & \;+CO_{2}+{\left[\mathrm{mimC}H_{2}\mathrm{CO}O^{-}\right]}^{+} \end{array}$$

(P1‐R_‐1_‐WH_cat_) $$\begin{array}{c} \mathrm{MEA}H^{+}+{[\mathrm{mimC}H_{2}\mathrm{CO}O^{-}]}^{+}\to \mathrm{MEA}+{\left[\mathrm{mimC}H_{2}\mathrm{COOH}\right]}^{+} \end{array}$$

In the catalysis pathway, P1‐WH_cat_‐MEA‐CO_2_, in presence of [mimCH_2_COOH]^+^, MEACOO^−^ decomposes into MEA and CO_2_ in one step without ZW formation. Its Gibbs free energies of activation are 4.7 kcal mol^−1^, much lower than that in P1‐WO_cat_‐MEA‐CO_2_ (12.7 kcal mol^−1^). And then, to put into the next catalytic cycle, the other absorption product MEAH^+^ would provide the proton to [mimCH_2_COO^−^]^+^ to regenerate the catalyst. The apparent activation energy for CO_2_ absorption and desorption in P1‐WH_cat_‐MEA‐CO_2_ are 13.8 and 11.6 kcal mol^−1^, respectively, which are 13.8 and 19.6 kcal mol^−1^ in the pathway without catalyst P1‐WO_cat_‐MEA‐CO_2_. Thus, [mimCH_2_COOH]^+^ can clearly facilitate P1‐WO_cat‐_MEA‐CO_2_ based CO_2_ capture pathway, with the P1‐WH_cat_‐MEA‐CO_2_ more preferable.

Also, energy profiles for CO_2_ absorption and desorption in P2‐R_1_ and P2‐R_2_, with and without the presences of [mimCH_2_COOH]^+^, were calculated and presented in Figure S11 (Supporting Information). The Gibbs free energy of activation for the catalytic CO_2_ absorption in P2‐R_1_‐WH_cat_‐MEA‐CO_2_‐H_2_O is 2.6 kcal mol^−1^, lower than 7.1 kcal mol^−1^ in the noncatalytic P2‐R_1_‐WO_cat_‐MEA‐CO_2_‐H_2_O. While the Gibbs free energy of activation for the other catalytic CO_2_ absorption, P2‐R_2_‐WH_cat_‐MEA‐CO_2_‐H_2_O, are 12.7 and 3.5 kcal mol^−1^, respectively, which is very close to 12.7 and 3.9 kcal mol^−1^ in the noncatalytic P2‐R_2_‐WO_cat_‐MEA‐CO_2_‐H_2_O. Thus, both noncatalytic P2‐R_1_‐WO_cat_‐MEA‐CO_2_‐H_2_O and the catalyzed P2‐R_1_‐WH_cat_‐MEA‐CO_2_‐H_2_O appear to be easier for CO_2_ capture with H_2_O pathway.

As shown in Figure S10 (Supporting Information), [mimCH_2_COOH]^+^ is more effective than MEA‐cat Complex in accelerating not only HCO_3_^−^ formation during CO_2_ absorption, but also HCO_3_^−^ decomposition into CO_2_ during CO_2_ desorption in P2‐R_1_‐WH_cat_‐MEA‐CO_2_‐H_2_O. In noncatalytic P2‐R_1_‐WO_cat_‐MEA‐CO_2_‐H_2_O, the Gibbs free energy of activation for CO_2_ desorption is 12.6 kcal mol^−1^, and with [mimCH_2_COOH]^+^ that for CO_2_ catalytic desorption in P2‐R_1_‐WH_cat_‐MEA‐CO_2_‐H_2_O slightly decrease to 11.5 kcal mol^−1^, as shown in Figure 6B. The participation of [mimCH_2_COOH]^+^ does not change the reaction pathway, however the apparent activation energy for catalytic CO_2_ absorption and desorption in P2‐R_1_‐WH_cat_‐MEA‐CO_2_ are 12.4 and 14.6 kcal mol^−1^, which are 15.7 and 17.9 for noncatalytic CO_2_ absorption and desorption in P2‐R_1_‐WO_cat_‐MEA‐CO_2_, respectively. Then, after MEAH^+^ transfer proton to [mimCH_2_COO^−^]^+^ and produce [mimCH_2_COOH]^+^ and MEA, the catalyst regenerates. Also, the regenerated MEA can be used for CO_2_ absorption in the subsequent cyclic CO_2_ capture.

The reactions in the desorption pathway with the use of [mimCH_2_COOH]^+^ are

(P2‐R_‐1,2_‐WH_cat_) $$\begin{array}{c} {\mathrm{HCO}}_{3}^{\;-}+{\left[\mathrm{mimC}H_{2}\mathrm{COOH}\right]}^{+}\to CO_{2}+H_{2}O\;+\;{\left[\mathrm{mimC}H_{2}\mathrm{CO}O^{-}\right]}^{+} \end{array}$$

(P2‐R_‐1,1_‐WH_cat_) $$\begin{array}{c} \mathrm{MEA}H^{+}+{\left[\mathrm{mimC}H_{2}\mathrm{CO}O^{-}\right]}^{+}\to \mathrm{MEA}+{\left[\mathrm{mimC}H_{2}\mathrm{COOH}\right]}^{+} \end{array}$$

Obviously, [mimCH_2_COOH]^+^ as a Brønsted acid or the core of the novel catalytic CO_2_ capture process, is necessary for initiating and driving P2‐R_1_‐WH_cat_, which further confirms the significant function of hydrolysis of [EMmim][NTf_2_] during CO_2_ absorption. The electrophilic characteristic of mim^+^ in [mimCH_2_COOH]^+^ can enhance the acidity of the —COOH in [mimCH_2_COOH]^+^ via inductive effect, which is desired for P2‐R_‐1,2_‐WH_cat_ or formation of CO_2_ and H_2_O as well as [mimCH_2_COO^−^]^+^. The stable intermediate ([mimCH_2_COO^−^]^+^) resulting from the inherent resonance effect existing in —COO^−^ is a conjugate base of [mimCH_2_COOH]^+^, which can quickly react with Brønsted acid, MEAH^+^ in P2‐R_‐1,1_‐WH_cat_ for realization of MEA regeneration. Furthermore, the essential part of P2‐R_‐1,2_‐WH_cat_ is to convert HCO_3_^−^ into CO_2_ and H_2_O, which is a well‐known rate limiting step in all the chemisorption‐based capture CO_2_ technologies. Thus, [EMmim][NTf_2_] with the desired Brønsted acidity is shown to be a highly effective homogenous organocatalyst for chemisorption‐based capture CO_2_ technologies.

## 3 Conclusions

The much quicker CO_2_ desorption kinetics at low temperature enabled with the use of the IL catalyst can significantly advance the development of a new generation of CO_2_ capture technology, from the perspectives of decreasing the parasitic penalty of these systems, capital investment, and environmental protection. The catalyst can make CO_2_ capture much less demanding for high quality energy, and thus widely available low‐temperature heat (e.g., those from solar collectors or waste heat) can be effectively used for CO_2_ capture, which will not only leads to a significant decrease in parasitic energy penalty, capital and operating costs, but also is beneficial to the elimination of the secondary environmental pollutant resulting from MEA degradation during high‐temperature CO_2_ desorption of conventional CO_2_ capture technologies. Therefore, [EMmim][NTf_2_] is a transcendent catalyst for CO_2_ capture technology.

## 4 Experimental Section

### Experimental Design—Synthesis of IL

The detailed synthesis information of the [EMmim][NTf_2_] catalyst is presented as follows, and the schematic diagram of the synthetic route is displayed in Figure S2 (Supporting Information). First, [EMmim][Br] was synthesized with the reaction between 1‐methylimidazole and methyl bromoacetate. In a typical experiment, methyl bromoacetate (0.155 mol) was added dropwise to a solution of 1‐methylimidazole (0.15 mol) in acetonitrile (150 mL) under nitrogen atmosphere. The mixture was continuously stirred at room temperature until it was thoroughly homogeneous, following by heating the mixture at 55 °C for 12 h. The resulting solution was evaporated under vacuum and washed with ethyl acetate repeatedly to remove excess methyl bromoacetate. [EMmim][Br] was obtained after evaporating the above solution under vacuum condition. The produced IL was dried under vacuum for at least 24 h before use (31.6 g, yield 89.6%). [EMmim][NTf_2_] was prepared from the metathesis reaction between [EMmim][Br] and [Li][NTf_2_]. In a typical procedure, [EMmim][Br] (0.06 mol) was mixed with equimolar amount of [Li][NTf_2_] in 20 mL water. Then the mixture was vigorously stirred for 4 h at room temperature. After that, the bottom phase was washed with deionized water repeatedly to remove the excess salt. The resulting solution was rotary evaporated under vacuum to obtain the final product [EMmim][NTf_2_]. The prepared IL was dried under vacuum for at least 24 h before use (23.1 g, yield 88.3%).

### Experimental Design—CO_2_ absorption–desorption Test

100 mL of 20 wt% MEA (99+%, product number: 11016‐7‐4L, Aldrich chemical company, Inc.) aqueous solution with the desired quantity of IL catalyst was employed for each test. The first run of CO_2_ absorption process was conducted at 30 °C and atmospheric pressure (0.78 bar at Laramie, Wyoming, USA, where the experiment was conducted), while the cyclic tests were conducted at 22 °C (room temperature). The simulated flue gas containing 10 vol% CO_2_, 10 vol% O_2_ and 80 vol% N_2_ with a total flow rate of 500 mL min^−1^ was bubbled into the 20 wt% amine solution. The outlet gas concentration was monitored with an inline gas analyzer (NDIR ZRE, California Analytical Instruments), and then recorded using a data recording unit. Absorption time for the first run test was 7800 s and that for each cyclic test was 1800 s. Upon the completion of the CO_2_ absorption experiment, the temperatures of the uncatalytic and catalytic MEA solutions were gradually heated to 85 °C. During the desorption process, N_2_ with a flow rate of 500 mL min^−1^ was used as the carrier gas. Desorption times were 1800 s for both first‐run and cyclic tests. The uncatalytic and catalytic solutions for Fourier‐transform infrared (FT‐IR) spectroscopy characterizations were collected under similar conditions except for different absorption or desorption times.

### Statistical Analysis—Characterizations

^1^H NMR and ^13^C NMR spectra were recorded on a Bruker spectrometer (400 MHz) in the solvent of DMSO. ESI‐MS spectrum was obtained by ultra‐high‐resolution electro‐spray time‐of‐flight mass spectrometry (Bruker microTOF II, Germany). FT‐IR spectra were collected using a Thermo Nicolet Magna‐IR 760 spectrometer with a resolution of 4 cm^−1^ by scanning 32 times from 4000 to 400 cm^−1^. Thermogravimetric analysis (TGA) of IL was obtained using a TA Instruments SDT Q600 apparatus with a heating ramp of 10 °C min^−1^ at the temperature of 20–600 °C with a nitrogen flow rate of 100 mL min^−1^.

### Statistical Analysis—Theoretical Studies

All of the calculations were performed with Gaussian09 package. Geometry optimization of all the minima and transition states involved was carried out at the B3LYP ^[^ ^31^, ^32^ ^]^ level with the 6–31G(d) basis set for C, H, O, N. Default convergence criteria were used. The vibrational frequency calculations were conducted at the same level of theory as geometry optimization to confirm whether each optimized structure is an energy minimum or a saddle point. For transition state, intrinsic reaction coordinate (IRC) analysis ^[^ ^33^ ^]^ was performed to verify that it connects the right reactants and products. The solvent effects were considered using the PCM model ^[^ ^34^, ^35^ ^]^ with the as‐phase‐optimized structures as the initial geometries.

## Conflict of Interest

The authors declare no conflict of interest.

## Supporting information

Supporting Information

## Data Availability

The data that support the findings of this study are available in the supplementary material of this article.
