# Supplementary material for: [EMmim][NTf2]—a Novel Ionic Liquid (IL) in Catalytic CO2 Capture and ILs’ Applications
Source: Adv Sci (Weinh). 2022 Nov 23;10(3):2205352. doi: 10.1002/advs.202205352 (PMC9875647; doi:10.1002/advs.202205352)
Supplement: Supplementary file 1 — Supporting Information [file ADVS-10-2205352-s001.pdf]

Supporting Information

[EMmim][NTf$_2$] – A Historic Ionic Liquid (IL) in Catalytic CO_2 Capture and ILs' Applications

Xin He[†], Yangyan Gao[†], Yunlei Shi, Xiaowen Zhang, Zhiwu Liang, Riguang Zhang, Xingfei Song, Qinghua Lai, Hertanto Adidharma, Fangqin Cheng, Armistead G Russell, Eric G. Eddings, Weiyang Fei, Maohong Fan[*], Shik Chi Edman Tsang[*], Jianji Wang[*]

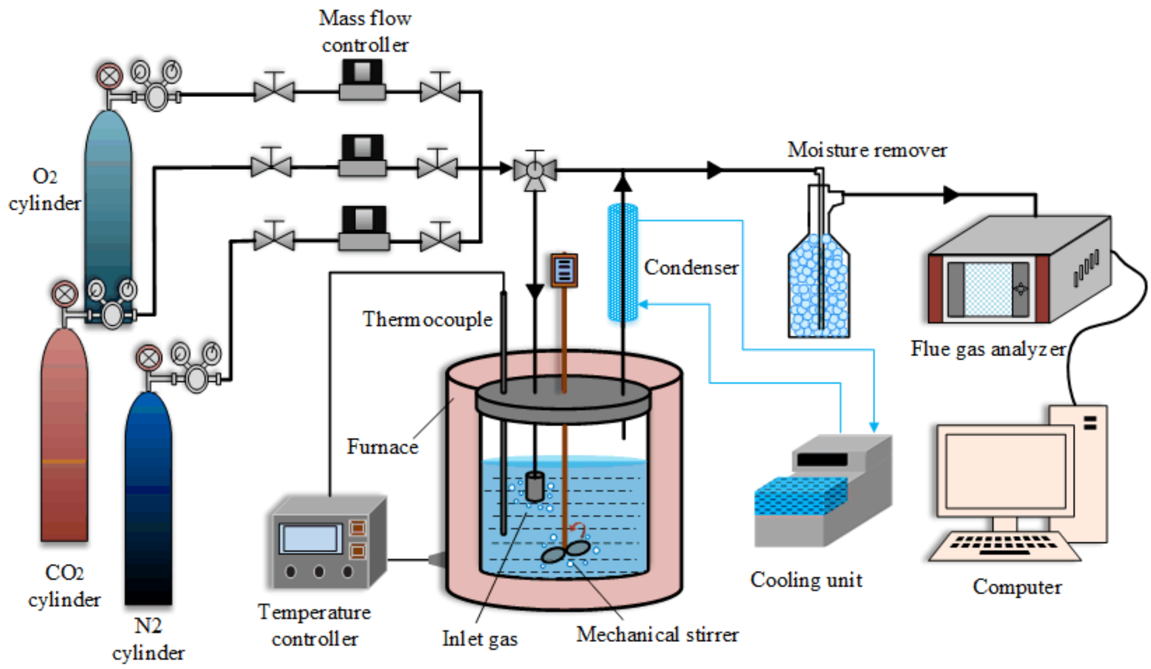

Figure S1. Schematic drawing of CO_2 absorption and desorption experimental setup. The inlet gas uses synthetic fule gas mixture with 10 vol% CO_2, 10 vol% O_2 and 80 vol% N_2.

A

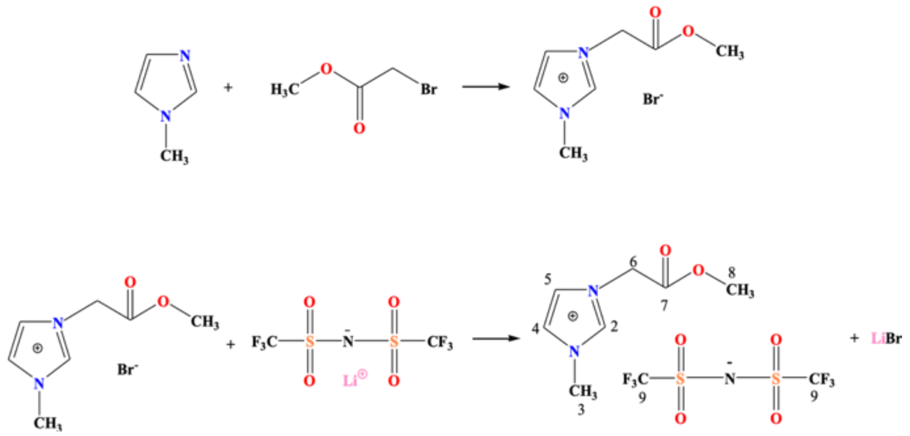
CH3
H3C
O
Br
Br-
N
F3C
CF3
S
Li
LiBr

B

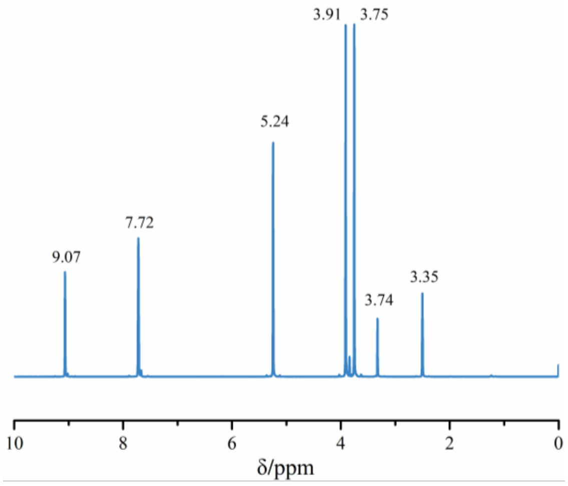
3.91
3.75
5.24
7.72
9.07
3.35
3.74
δ/ppm

C

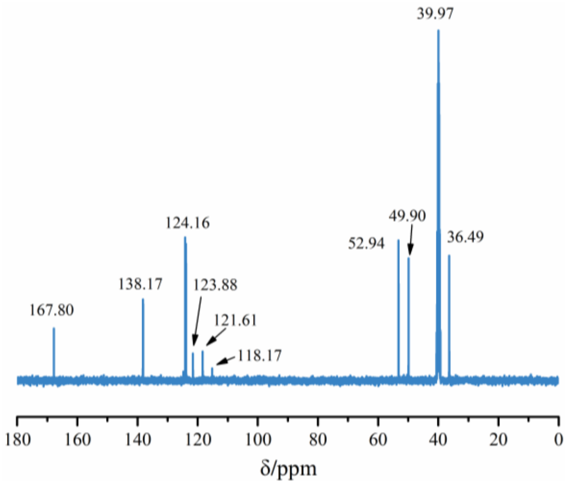
39.97
124.16
49.90
52.94
36.49
138.17
123.88
167.80
121.61
118.17
δ/ppm

D

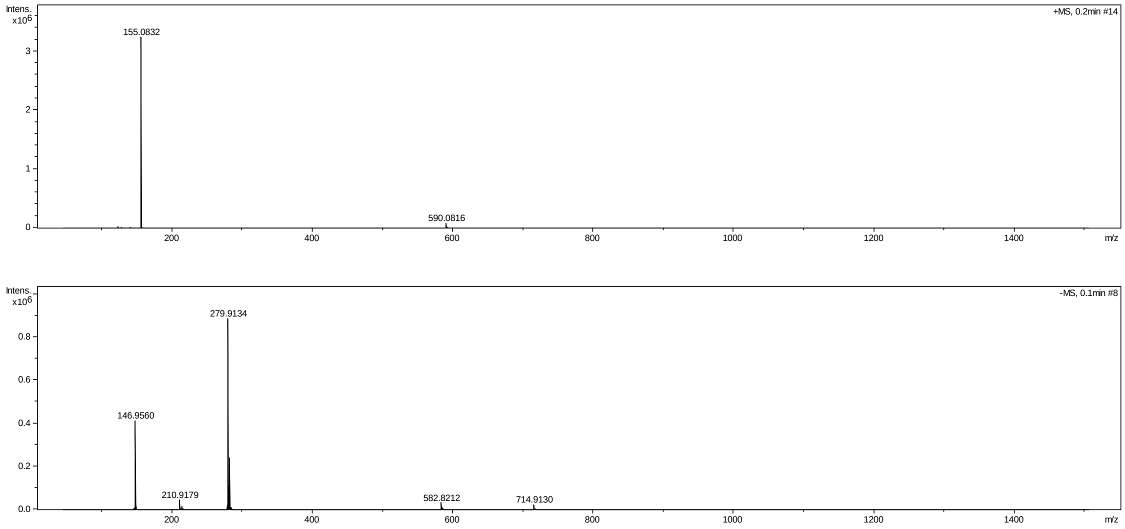
Intens.
x10^6
+MS, 0.2min #14
155.0832
590.0816
m/z
-MS, 0.1min #8
279.9134
146.9560
210.9179
582.8212
714.9130

E

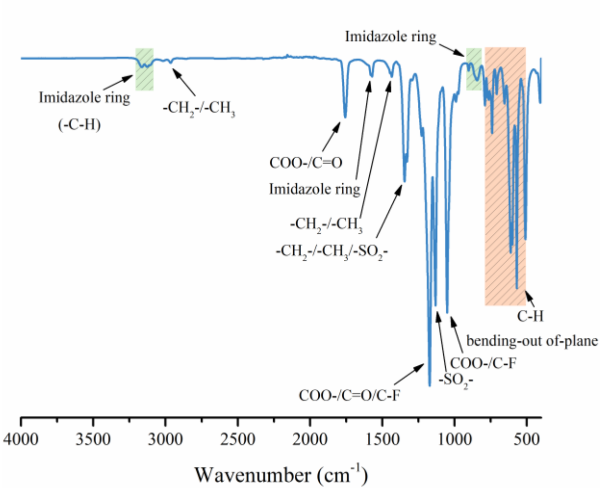

F

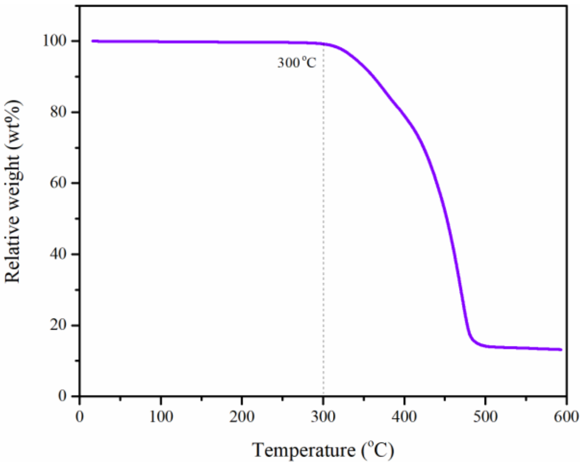

Figure S2. Information of the synthesized IL [EMmim][NTf_2]. (**A**) Schematic diagram of the synthetic route of IL [EMmim][NTf_2]; (**B**) ^1H NMR spectrum of [EMmim][NTf_2]. ^1H NMR (Bruker spectrometer, 400 MHz, d6-DMSO) data: δ 9.07 (s, 1H, H2), 7.72 (s, 2H, H4 and H5), 5.24 (s, 2H, H6), 3.91 (s, 3H, H8), 3.75 (s, 3H, H3); (**C**) ^{13}C NMR spectrum of [EMmim][NTf_2]. ^{13}C NMR (Bruker spectrometer, 400 MHz, d6-DMSO) data: δ 36.49 (C3), 49.90 (C8), 52.94 (C6), 118.17, 121.61 (C9), 123.88, 124.16 (C4, C5), 138.17 (C2), 167.80 (C7); (**D**) ESI-MS spectrum of [EMmim][NTf_2]. Upper: m/z = 155.1 for $[EMmim]^+$; Lower: m/z = 279.9 for $[NTf_2]^-$. ESI-MS spectrum was obtained by ultra-high-resolution electro-spray time-of-flight mass spectrometry (Bruker micro TOF II, Germany); (**E**) FT-IR spectrum of [EMmim][NTf_2], collecting with a Thermo Nicolet Magna-IR 760 spectrometer with a resolution of 4 cm^{-1} by scanning 32 times from 4000 to 400 cm^{-1}; (**F**) TG curve of [EMmim][NTf_2], obtained with a heating ramp of 10 °C min^{-1} within 20-600 °C with a nitrogen flow rate of 100 mL min^{-1}. Results show that IL can be stable at as high as 300 ^oC.

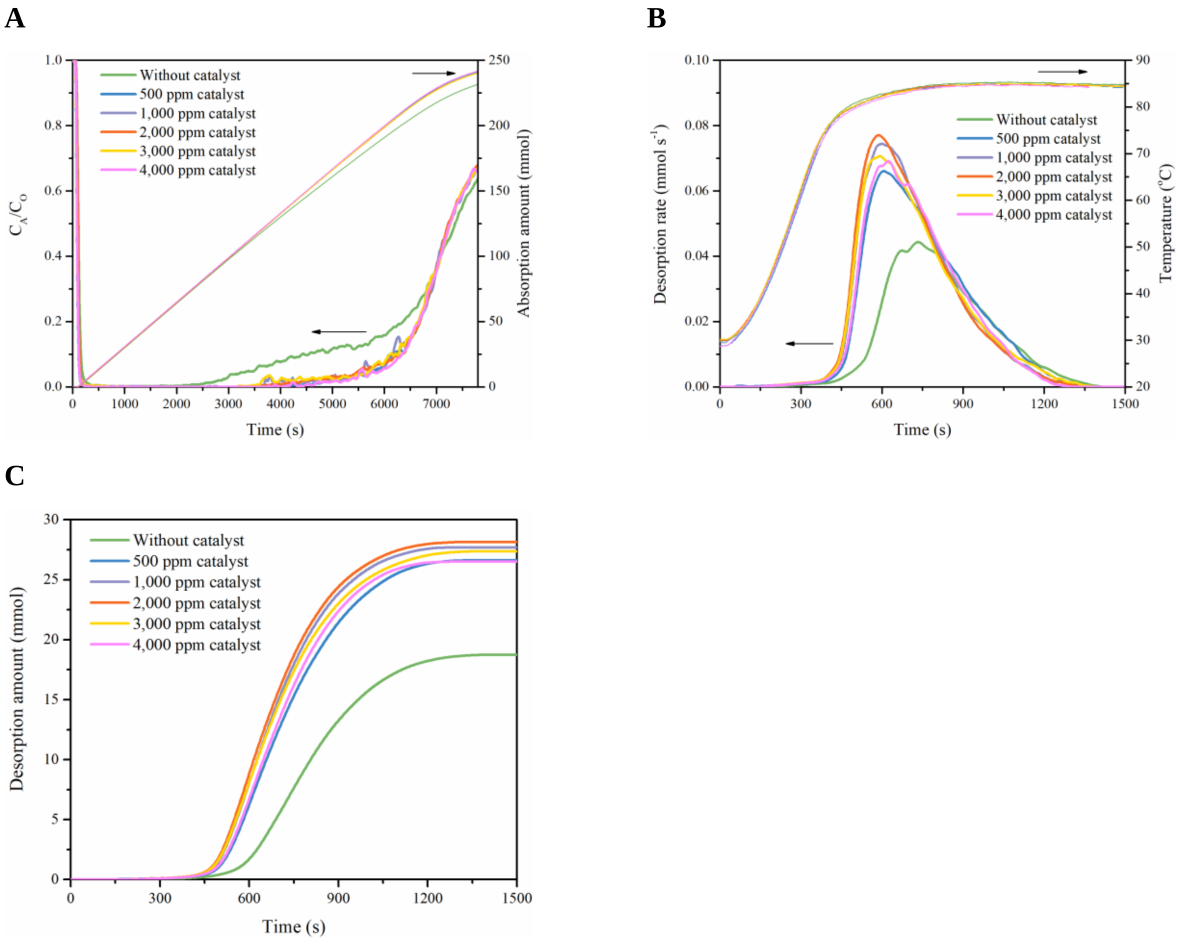

Figure S3. Catalytic effects of [EMmim][NTf$_2$] dosage on the CO_2 absorption and desorption performances- (**A**) CO_2 absorption profiles of 20 wt% MEA sorbent; (**B**) CO_2 desorption rates of 20 wt% MEA sorbent; (**C**) Total CO_2 desorption amounts. Absorption conditions: total mass of solution: 100g; MEA concentration: 20 wt%; inlet gas flow rate: 500 mL min^{-1}; inlet gas composition: 80 vol% N_2, 10 vol% CO_2 and 10 vol% O_2; absorption time: 7800 s. Desorption conditions: total mass of solution: 100 g; MEA concentration: 20 wt%; carrier gas (N_2) flow rate: 500 mL min^{-1}; temperature: 85 ^{o}C; time: 1800 s. (**B**) and (**C**) show the time length of 1500 s because the desorption rate have dropped to zero for all solutions at 1500 s, indicating that no more CO_2 could be desorbed from the solutions after 1500 s under the above mentioned experimental conditions.

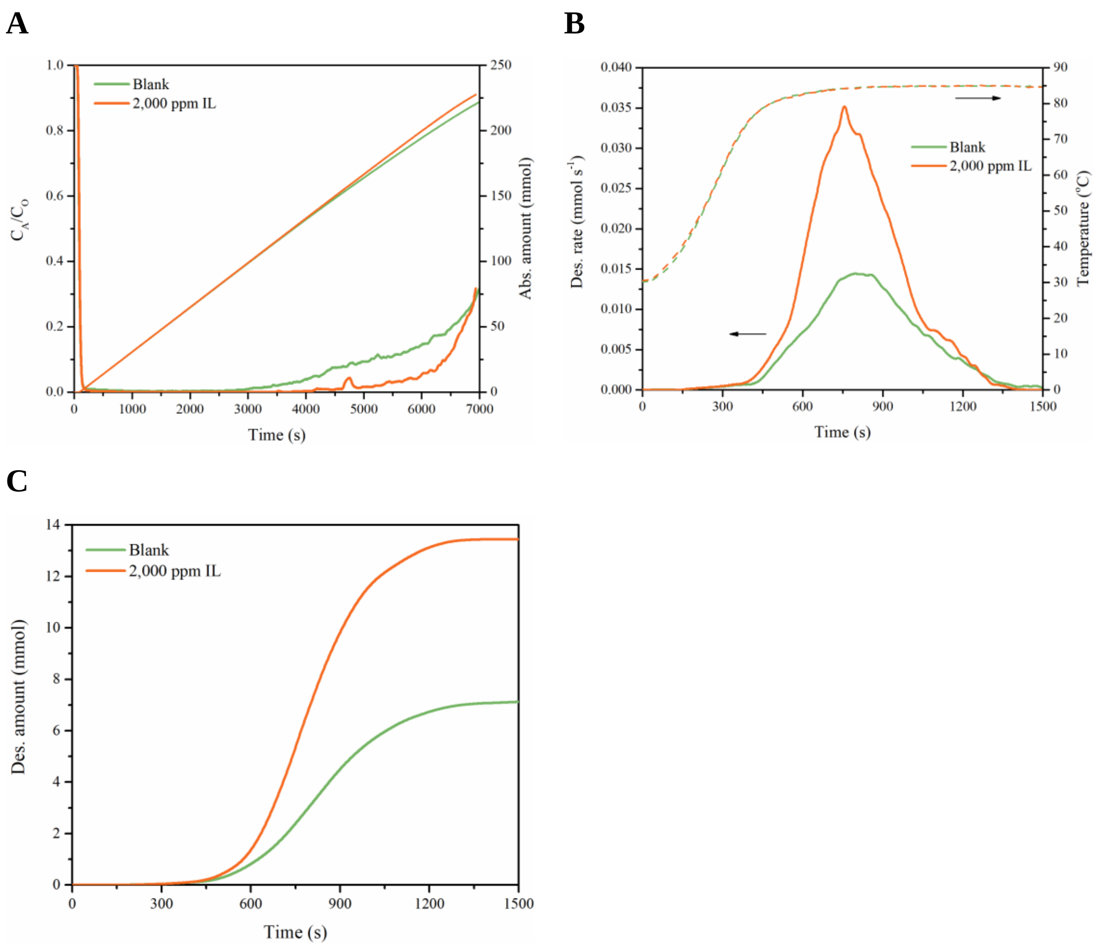

Figure S4. CO_2 absorption and desorption curves with and without catalyst when the absorption processes were terminated within 7000s with the same outlet CO_2 concentrations (3.2 vol%)- (**A**) CO_2 absorption profiles of 20 wt% MEA sorbent; (**B**) CO_2 desorption rates of 20 wt% MEA sorbent; (**C**) Total CO_2 desorption amounts. Absorption conditions: total mass of solution: 100g; MEA concentration: 20 wt%; gas flow rate: 500 mL min^{-1}; gas composition: 80 vol% N_2, 10 vol% CO_2 and 10 vol% O_2; absorption time: 7000 s. Desorption conditions: total mass of solution: 100 g; MEA concentration: 20 wt%; carrier gas (N_2) flow rate: 500 mL min^{-1}; temperature: 85 ^{o}C; time: 1800 s. (**A**) and (**B**) show the time length within 1500 s because the desorption rate have dropped to zero for both uncatalyzed and catalyzed solutions at 1500 s, indicating that no more CO_2 was desorbed from the uncatalytic and catalytic solutions after 1,500 s under the above mentioned experimental conditions.

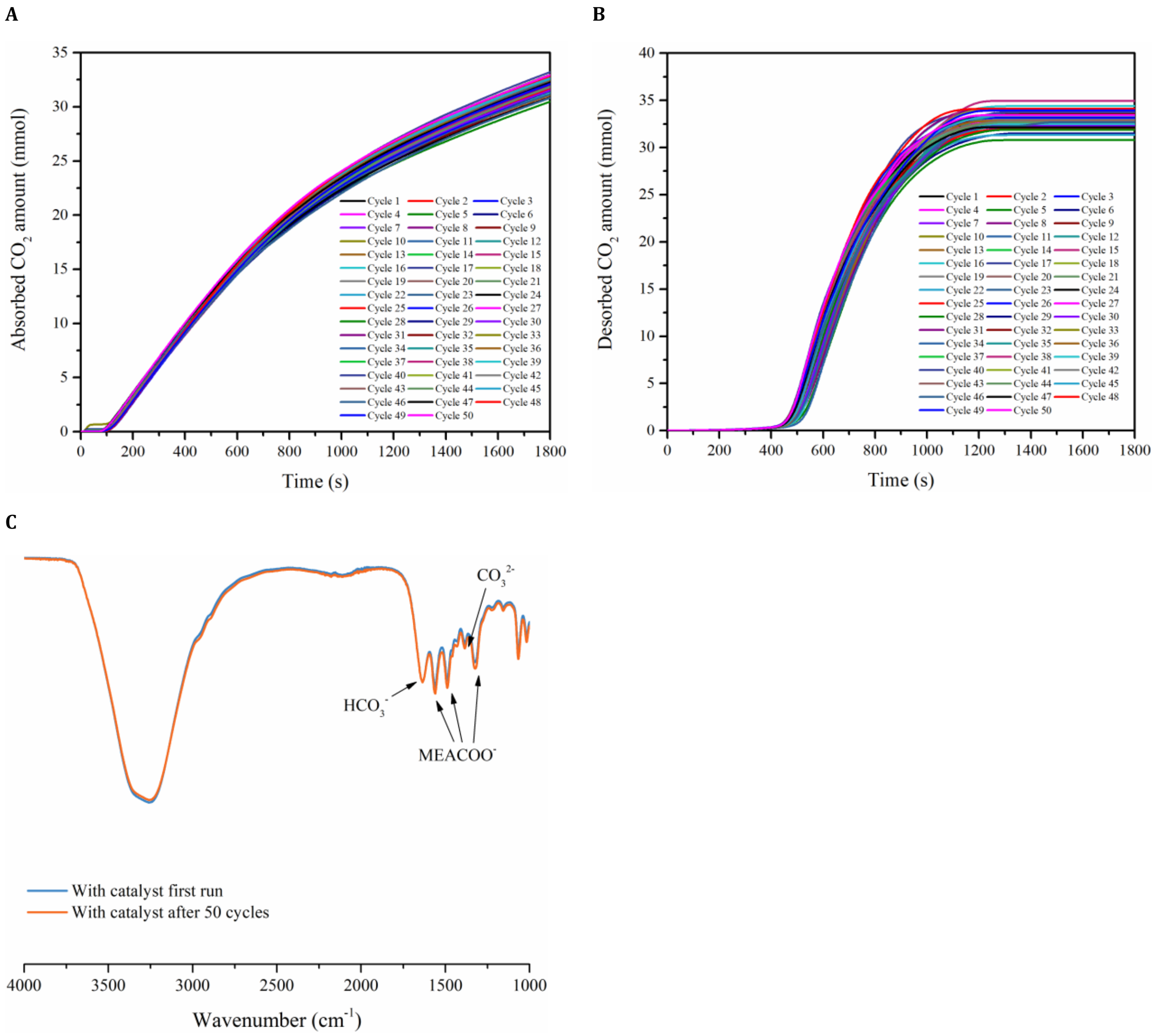

Figure S5. Information of the cyclic tests. (A) Quantities of absorbed and (B) desorbed CO_2 with the presence of [EMmim][NTf$_2$] catalyst during 50 cyclic tests; (C) Comparison of FT-IR spectra of 20 wt% MEA solution with [EMmim][NTf$_2$] catalyst after first run and 50 cyclic tests. HCO_3^-, CO_3^{2-} and $MEACOO^-$ are generated during CO_2 absorption process. No change was observed for the structure of MEA molecules after 50 cyclic tests. [Absorption conditions: 100 g 20 wt% MEA solution with 2,000 ppm catalyst; composition of inlet gas: 10 vol% CO_2, 10 vol% O_2, and 80 vol% N_2; flow rate of inlet gas: 500mL min^{-1}; time: 1,800 s. Desorption conditions: flow rate of carrier gas (N_2): 500 mL min^{-1}; temperature: 85 °C; time: 1,800s].

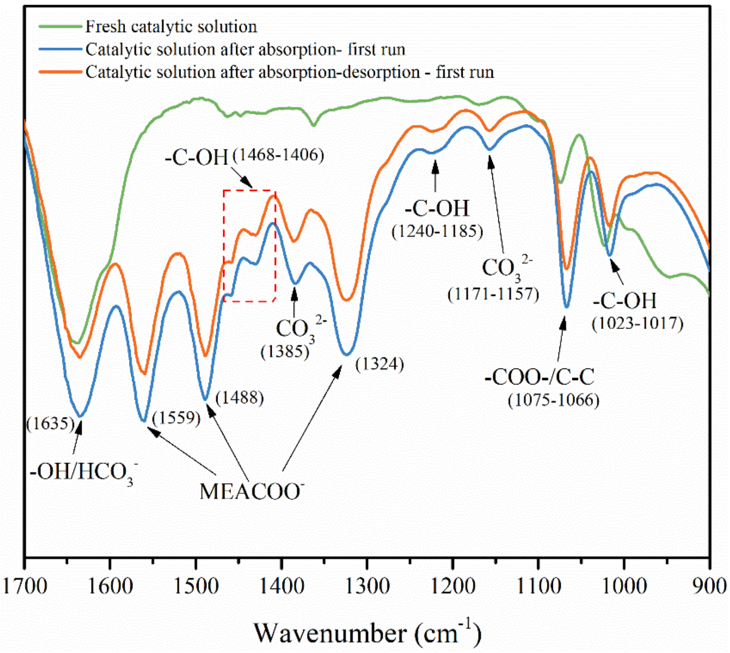

Figure S6. FT-IR spectra of the fresh catalytic solution, catalytic solution after 1st absorption and 1st cyclic absorption-desorption. Peak at 1023-1017 cm^{-1} is assigned to -C-OH, belonging to MEA (-CH_2-OH) and HCO_3^- (-COOH) after absorption. Peak at 1075-1066 cm^{-1} is assigned to -COO-/C-C, belonging to MEA (-CH_2-CH_2-), and HCO_3^- (-COO-) resulting from absorption. Peaks at 1171-1157 cm^{-1} and 1385 cm^{-1} are assigned to CO_3^{2-} resulting from absorption. Peaks at 1240-1185 cm^{-1} and 1468-1406 cm^{-1} are assigned to -C-OH, belonging to HCO_3^- (-C-OH) resulting from absorption. Peaks at 1559, 1488, 1324 cm^{-1} are assigned to -COO- in MEACOO- resulting from absorption. Peak at 1635 cm^{-1} is assigned to HCO_3^- or -OH, which is also a signature peak of water FT-IR.

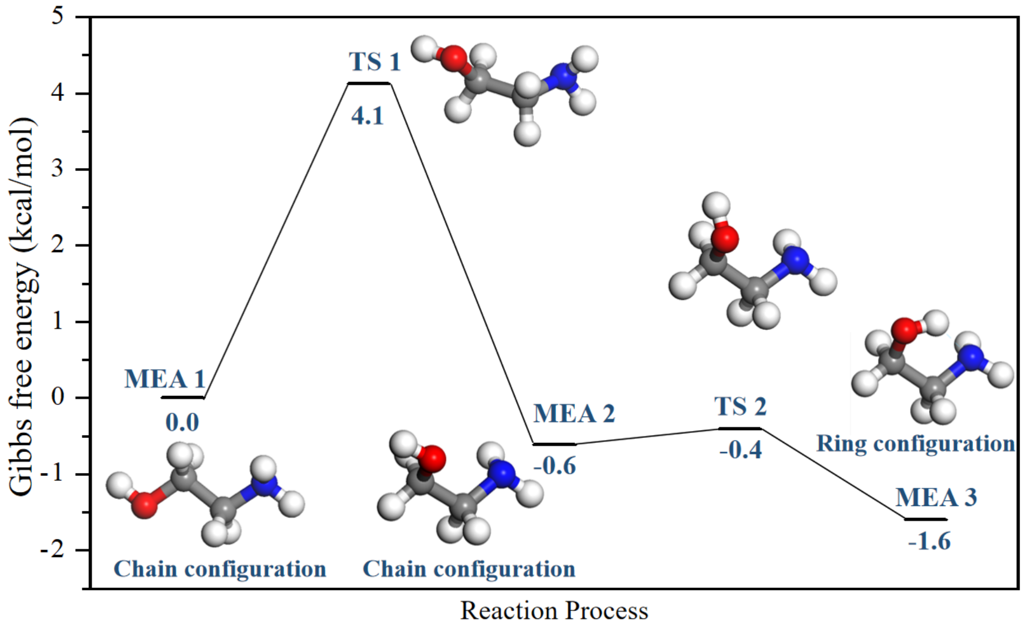

Figure S7. The optimized geometries associated with transformations between different MEA configurations. The stability order of MEA is Ring configuration > Open-Ring configuration > Chain configuration. However, the activation energies for transformation between each other are moderate, indicating that these three configurations could exist at the same time. Ring configuration, the most stable structure, was applied in the calculations.

A

B

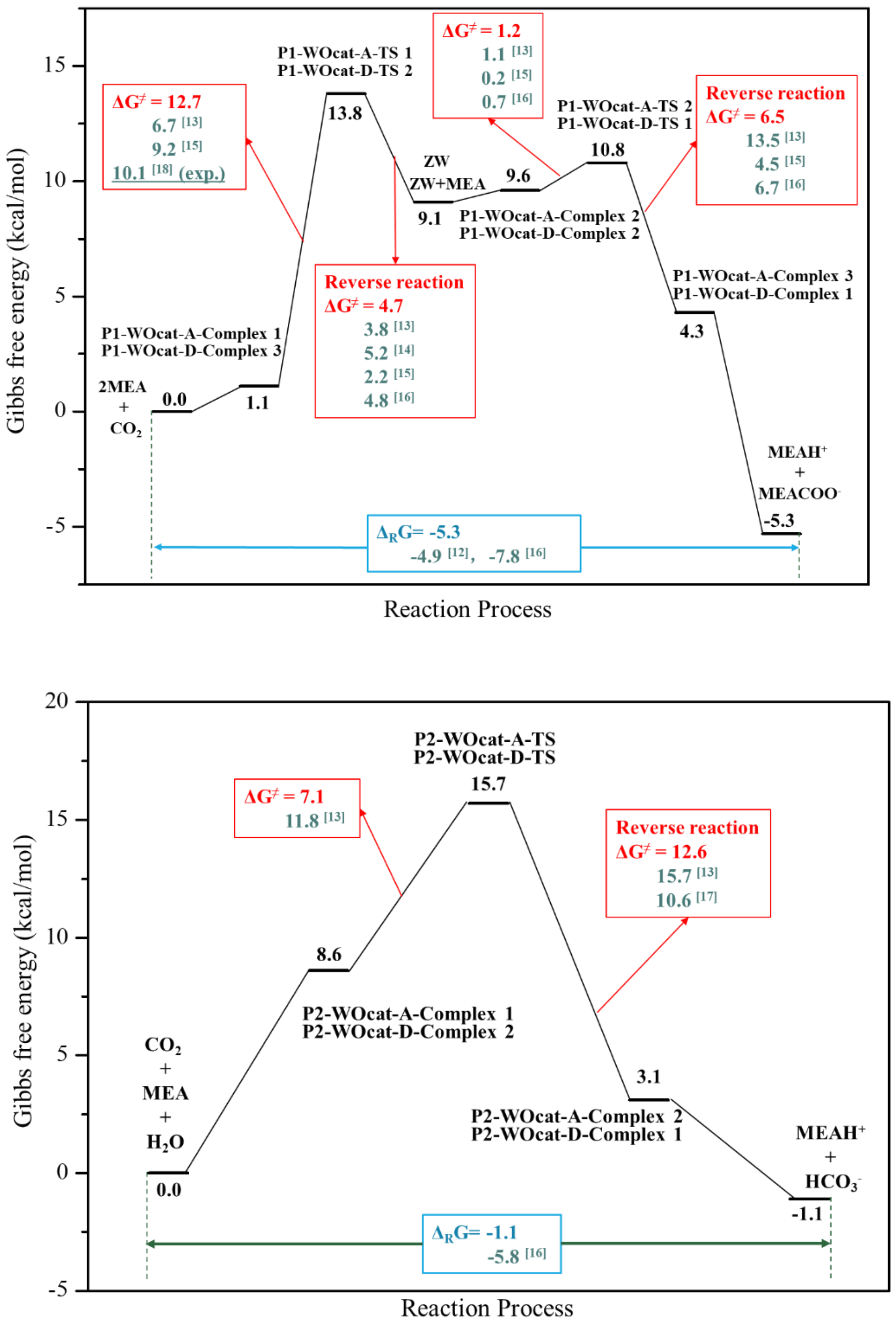

Figure S8. The relative Gibbs free energies of activation and reaction comparisons. The Gibbs free energy of activation and reaction for CO_2 absorption and desorption in (**A**) ZW mechanism (P1) and (**B**) bicarbonate mechanism (P2-R1) were compared with those reported in literature, and it is found that the values are relatively close, which proves that the calculation method is reliable.

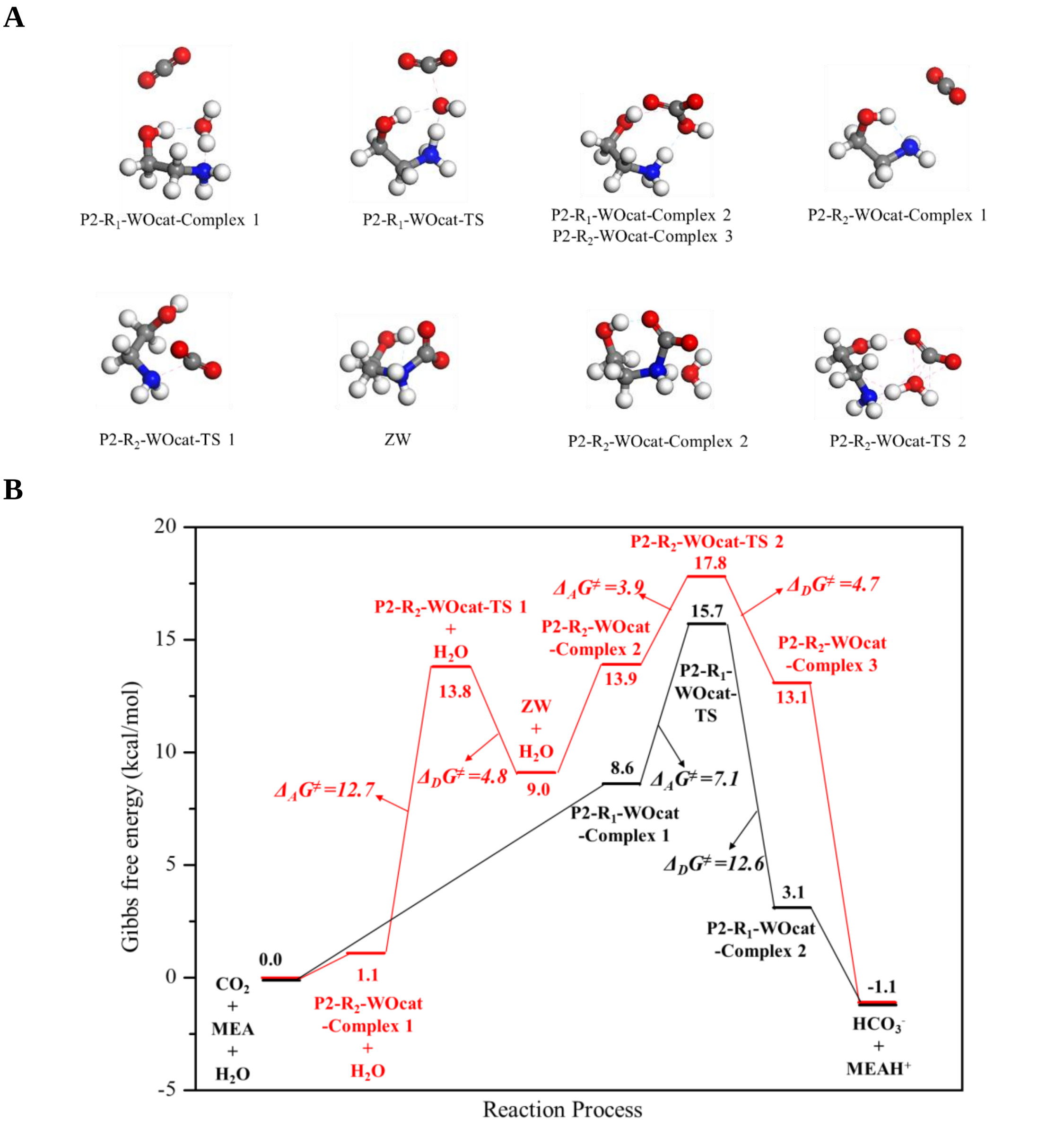

Figure S9. Comparisons of the relative Gibbs free energies of activation in P2-R_1 and P2-R_2 for H_2O-MEA-CO_2 system. (**A**) The optimized geometries associated with pathways for P2-R_1 and P2-R_2. (**B**) Gibbs free energy profiles for the possible pathways for P2-R_1 and P2-R_2. $\Delta_A G^{\neq}$ and $\Delta_D G^{\neq}$ are used to represent the Gibbs free energies of activation for the absorption and desorption process, respectively. In P2-R_1, H_2O, MEA and CO_2 interact with each other at the same time, and produce HCO_3^- and $MEAH^+$ in one step. The Gibbs free energy of activation for adsorption and desorption in P2-R_1 are 7.1 kcal/mol and 12.6 kcal/mol, respectively. The apparent activation energy for CO_2 absorption and desorption in P2-R_1 are 15.7 and 16.8 kcal/mol, respectively. In P2-R_2, H_2O participates later in the reaction after ZW was formed from MEA and CO_2, which is the same as P1-R_1, and then ZW react with H_2O to produce HCO_3^- and $MEAH^+$. The Gibbs free energy of activation for the

second step is 3.7 kcal/mol. The apparent activation energy for CO_2 absorption and desorption in P2-R_2 are 17.8 and 18.9 kcal/mol, respectively.

A

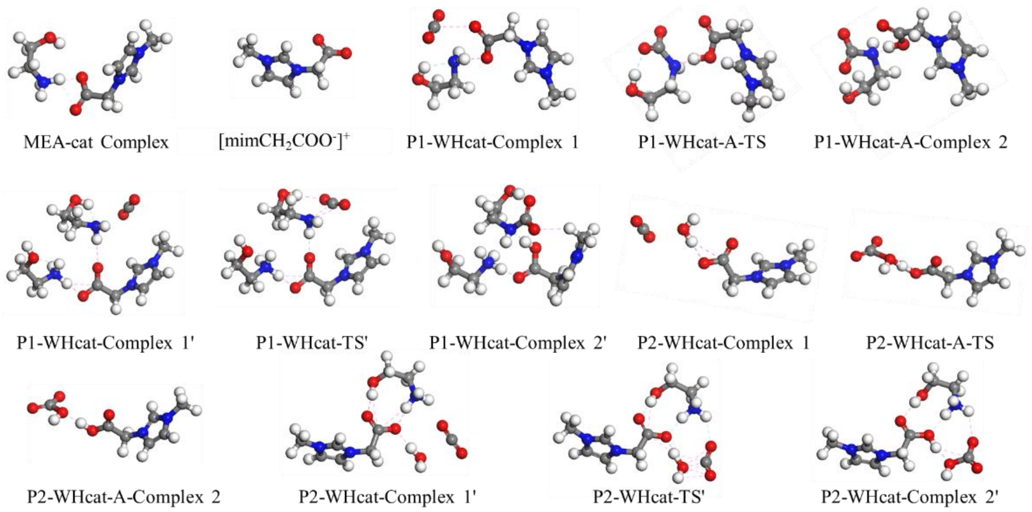

B

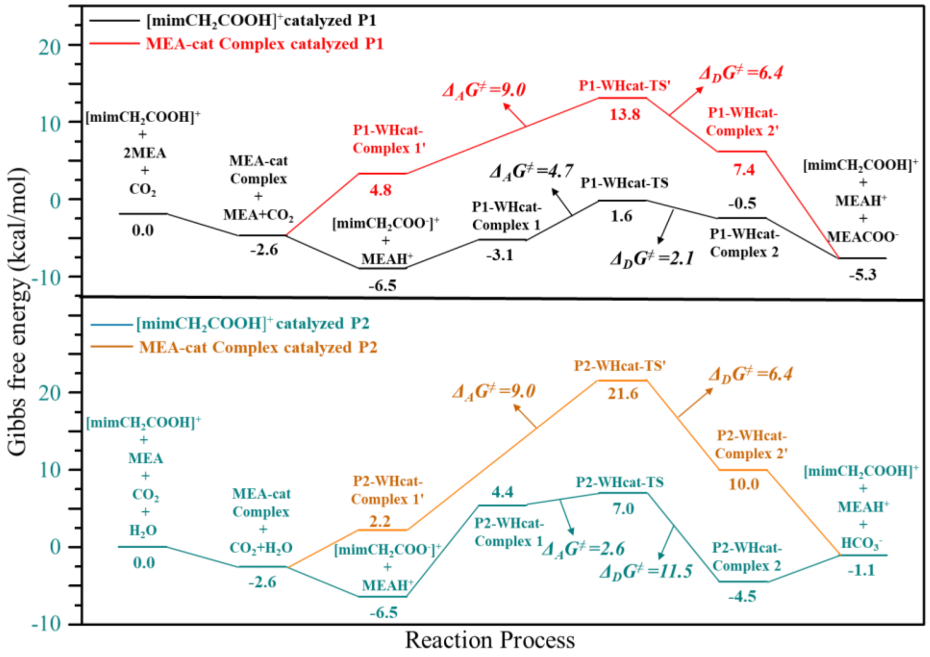

Figure S10. Catalysis ability comparisons between MEA-cat Complex and $[mimCH_2COOH]^+$. There is a strong interaction between MEA and $[mimCH_2COOH]^+$, producing MEA-cat Complex. Thus, $[mimCH_2COOH]$ could act as catalysts as MEA-cat Complex, or as individual component separated from MEA. After comparison, the individual $[mimCH_2COOH]^+$ is preferred to be the catalyst of the process. (**A**) Optimized geometries associated with the possible catalyzed pathways for MEA-CO_2 and H_2O-MEA-CO_2. (**B**) Gibbs free energy profiles for the

possible catalyzed pathways for MEA-CO_2 and H_2O-MEA-CO_2. $\Delta_A G^{\neq}$ and $\Delta_D G^{\neq}$ are respectively used to represent the Gibbs free energies of activation for the absorption and desorption process.

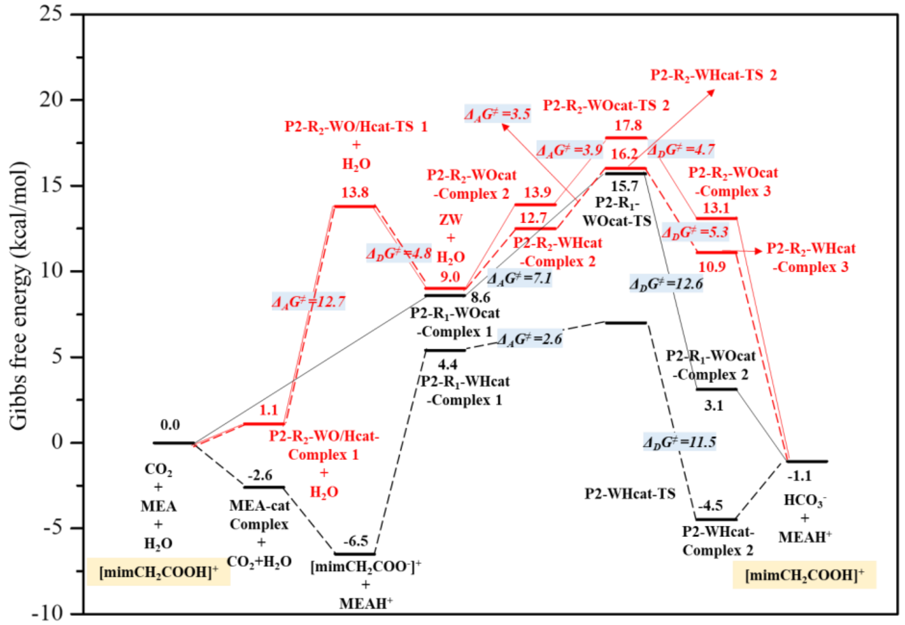

Figure S11. Comparisons of the relative Gibbs free energies of activation in P2-R1 and P2-R2, with and without [mimCH$_2$COOH]$^+$.
